# Idiopathic Cervical Transverse Myelitis Presenting Predominantly With Lower Limb Weakness and Autonomic Dysfunction: A Report of a Rare Case

**DOI:** 10.7759/cureus.84286

**Published:** 2025-05-17

**Authors:** Umer Farooq, Muhammad Hamza Zamir, Farhan Akbar, Ali Bilal

**Affiliations:** 1 Internal Medicine, Fauji Foundation Hospital Rawalpindi, Rawalpindi, PAK; 2 Surgery, Nishtar Medical University, Multan, PAK

**Keywords:** cervical spine, corticosteroid therapy, idiopathic, mri diagnosis, neurological deficits, spinal cord inflammation, transverse myelitis, urinary incontinence

## Abstract

Transverse myelitis (TM) is a rare neuroinflammatory disorder that can lead to significant morbidity if not promptly diagnosed and treated. We report the case of a 40-year-old female who presented with progressive bilateral lower limb weakness and urinary incontinence over a two-month period. Neurological examination revealed upper motor neuron signs and a defined sensory level, raising suspicion of a spinal cord pathology. MRI of the cervicothoracic spine showed T2- and Short Tau Inversion Recovery (STIR)-weighted hyperintense lesions extending from C3 to C6, with mild cord expansion, suggestive of an inflammatory demyelinating process consistent with transverse myelitis. An extensive laboratory evaluation was unremarkable, and differential diagnoses such as multiple sclerosis, neuromyelitis optica, and sarcoidosis were excluded. Cerebrospinal fluid (CSF) analysis was not performed, representing a diagnostic limitation. With no identifiable infectious or autoimmune etiology, a diagnosis of idiopathic cervical transverse myelitis was made. The patient received high-dose intravenous corticosteroids followed by structured physiotherapy, resulting in marked clinical improvement within one week. This case underscores the importance of early recognition and intervention in atypical TM presentations, especially those involving the cervical spine, and demonstrates the potential for favorable outcomes with timely, individualized treatment.

## Introduction

Transverse myelitis (TM) is a rare, immune-mediated inflammatory disorder of the spinal cord, characterized by acute or subacute motor, sensory, and autonomic dysfunction [1]. TM may occur as an idiopathic condition or secondary to infections, autoimmune diseases, malignancies, or post-vaccination responses. Idiopathic cases account for approximately 15%-30% of all TM cases, while the remainder are attributed to identifiable underlying etiologies [2]. The global annual incidence of TM is estimated at 1.34 to 4.6 cases per million people, with peaks in the second and fourth decades of life [2].

The thoracic spinal cord is the most frequently involved site in TM, accounting for nearly 70% of cases, followed by the cervical and lumbar regions [3]. Cervical involvement, although less common, is clinically significant due to its potential to affect a larger portion of the spinal cord and lead to more extensive neurological deficits. Moreover, cervical TM may present atypically, especially when lower limb weakness or autonomic dysfunction occurs in the absence of upper limb or sensory complaints, leading to diagnostic uncertainty.

In this report, we present the case of a 40-year-old female diagnosed with idiopathic cervical transverse myelitis who presented with isolated lower limb weakness and urinary incontinence, an uncommon symptom distribution for cervical lesions. This report aims to highlight the diagnostic challenges of idiopathic cervical TM with atypical symptomatology and reinforce the importance of early corticosteroid therapy and individualized treatment strategies in optimizing outcomes.

## Case presentation

A 40-year-old female with no significant comorbidities presented to the neurology outpatient department of a tertiary care hospital with a two-month history of progressive bilateral lower limb weakness and urinary incontinence. She was in her usual state of health when she initially noticed mild weakness in the lower limbs, which progressed over several weeks from difficulty in walking to complete inability to stand or ambulate independently. The urinary incontinence developed concurrently and was described as involuntary loss of urine, often resulting in bedwetting during sleep. The patient denied any history of trauma, fever, recent infection, headache, photophobia, vomiting, seizures, or bowel disturbances. There were no complaints suggestive of upper limb weakness or cranial nerve involvement.

The patient's past medical history was notable only for pulmonary tuberculosis 20 years ago, for which she completed the full course of anti-tubercular therapy. She had no known chronic illnesses, autoimmune diseases, or recent vaccinations. Family history was non-contributory. She was a housewife residing in a rural, non-cemented house with poor sanitation and reported consumption of untreated lake water. She had six children, all delivered via spontaneous vaginal delivery. There was no history of recent childbirth or postpartum complications.

On general examination, the patient was alert and oriented with a Glasgow Coma Scale (GCS) score of E4V5M6 (15/15). Mild pallor was observed, and vital signs were within normal limits. Neurological examination revealed increased muscle tone in all four limbs, more pronounced in the lower extremities. Despite hypertonia and brisk reflexes in the upper limbs, motor strength in the upper limbs was preserved, with no reported functional deficits or complaints. The power was graded as 4/5 in the lower limbs, while the upper limbs exhibited full strength (5/5) on the Medical Research Council (MRC) scale, confirming isolated motor weakness in the lower extremities. Deep tendon reflexes were brisk in the upper limbs and exaggerated in the lower limbs. Bilateral plantar reflexes were extensor (positive Babinski sign). Muscle wasting was more evident in the lower limbs. Sensory examination showed reduced sensation to all modalities below the sternal notch down to the level just above the umbilicus, corresponding to an approximate sensory level at T6. Cranial nerves were intact, and no signs of meningeal irritation were present. Systemic examination, including respiratory, cardiovascular, and gastrointestinal systems, was unremarkable.

As part of the diagnostic evaluation, routine laboratory investigations were conducted along with targeted testing to rule out common secondary causes of transverse myelitis. Serum vitamin B12 levels were within normal limits. Infectious screening, including HIV, syphilis (Venereal Disease Research Laboratory (VDRL) test), and human T-lymphotropic virus type 1 (HTLV-1) antibodies, was negative. An autoimmune panel, including antinuclear antibody (ANA), aquaporin-4 IgG, and myelin oligodendrocyte glycoprotein antibody (MOG-Ab), returned negative results. Cerebrospinal fluid (CSF) analysis was not performed due to logistical limitations; however, the combination of clinical features and characteristic MRI findings supported a diagnosis of idiopathic transverse myelitis after exclusion of secondary causes.

Based on the clinical presentation, a provisional diagnosis of transverse myelitis was made. Guillain-Barré syndrome (GBS) was considered as a differential diagnosis; however, the presence of a defined sensory level and upper motor neuron signs made TM more likely. The absence of antecedent infection, ascending weakness, and areflexia further argued against GBS. Baseline laboratory investigations were conducted to rule out infectious and metabolic causes. Results are shown in Tables 1, 2.

**Table 1:** Hematological and biochemical investigations. WBC: white blood cell count, RBC: red blood cell count, MCV: mean corpuscular volume, MCH: mean corpuscular hemoglobin, MCHC: mean corpuscular hemoglobin concentration, ALT: alanine aminotransferase, AST: aspartate aminotransferase, ALP: alkaline phosphatase.

Test	Result	Reference Range
WBC count (×10⁹/L)	10.9	4-11
Hemoglobin (g/dL)	10.5	13-18
RBC count (×10¹²/L)	4.57	3.8-5.2
MCV (fL)	70.9	77-95
MCH (pg)	22.9	26-32
MCHC (g/dL)	32.3	32-36
Platelets (×10⁹/L)	346	150-400
Neutrophils (%)	47.2	40-80
Lymphocytes (%)	33.8	20-40
Eosinophils (%)	10.1	1-6
Creatinine (mg/dL)	0.4	0.5-0.9
Urea (mg/dL)	15.1	10-50
Sodium (mmol/L)	142.9	135-145
Potassium (mmol/L)	2.41	3.5-5
ALT (U/L)	24	<40
AST (U/L)	25.6	<40
ALP (U/L)	75.7	40-120
Bilirubin total (mg/dL)	0.25	0.3-1.2

**Table 2:** Urine analysis and coagulation profile. pH: urine acidity/alkalinity (potential of hydrogen), WBCs (urine): white blood cells in urine, RBCs (urine): red blood cells in urine, INR: international normalized ratio, APTT (s): activated partial thromboplastin time in seconds.

Test	Result	Reference Range
Specific gravity	1.015	1.005-1.030
pH	6.5	4.6-8.0
WBCs (urine)	Many	2-5/hpf
RBCs (urine)	2/hpf	<3/hpf
Protein, glucose, ketones	Nil	Negative
Prothrombin time (s)	14.9	≤13
INR	1.24	0.9-1.3
APTT (s)	33	≤31

There was no leukocytosis, and inflammatory markers (ESR and CRP, if tested) were within normal limits, reducing the suspicion of an infectious etiology. Hypokalemia and mild anemia were corrected with electrolyte replacement and iron supplementation, respectively.

**Figure 1:**
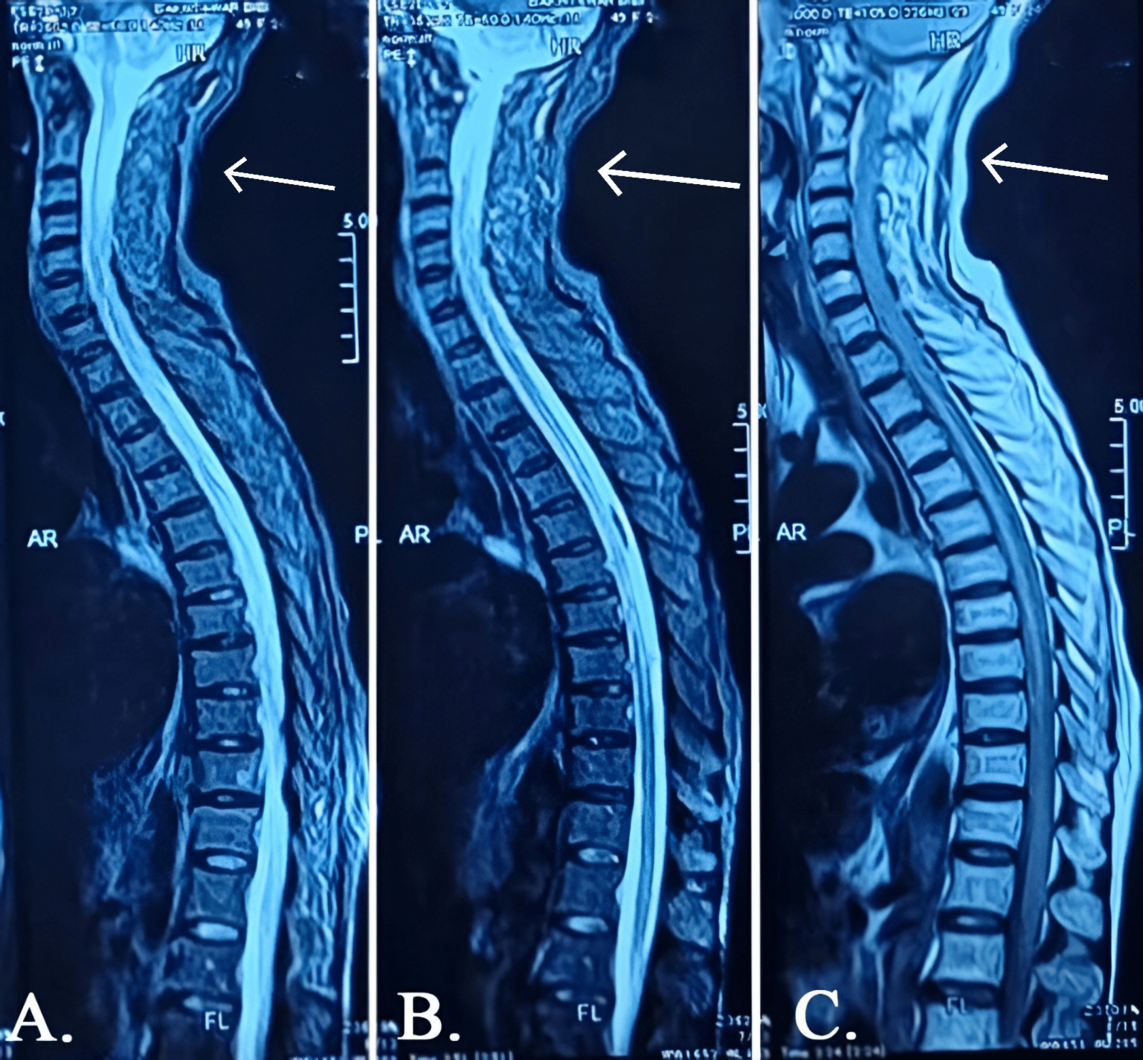
The MRI shows focal expansion of the cervical spinal cord with T2-/STIR-weighted hyperintense signals extending from C3 to C6, with the lesion indicated by arrows in panels A, B, and C. STIR: Short Tau Inversion Recovery, AR: anterior root, FL: foramen left, PL: posterior longitudinal, HR: herniated region.

An MRI of the cervicothoracic spine was performed to confirm the diagnosis. The imaging showed focal expansion of the cervical spinal cord with hyperintense signals on T2- and Short Tau Inversion Recovery (STIR)-weighted sequences extending from C3 to C6, measuring approximately 4-7 cm in length, as depicted in Figure 1. Additionally, there was evidence of myelomalacia and thinning of the dorsal spinal cord with a prominent central canal. These findings are consistent with a diagnosis of transverse myelitis, with cervical segment involvement. No mass lesions, compressive pathology, or vertebral instability was identified.

The patient was admitted and started on high-dose intravenous methylprednisolone at a dose of 1 g/day for five consecutive days. Concurrently, supportive care and physical rehabilitation were initiated, with a focus on lower limb strength, bladder training, and passive range-of-motion exercises. Given her preserved limb power (4/5) and early signs of improvement, plasma exchange and intravenous immunoglobulin were not deemed necessary. The patient responded well, showing progressive improvement in motor strength and sphincter control over the course of one week.

She was discharged on a tapering course of oral corticosteroids and scheduled for outpatient neurology and physiotherapy follow-up. At a three-week review, the patient reported significant functional improvement, was able to ambulate with minimal support, and had regained voluntary bladder control. No new neurological symptoms were reported, and no adverse effects from corticosteroids were observed. Written informed consent was obtained from the patient for the educational and academic use of her clinical case.

## Discussion

This case illustrates an uncommon presentation of transverse myelitis (TM) in a 40-year-old female with focal involvement of the cervical spinal cord. TM is a neuroinflammatory disorder that may present across all age groups, with incidence peaks in the second and fourth decades of life. While the thoracic spine is most frequently involved, accounting for the majority of TM cases, cervical involvement is less common but clinically significant due to its potential for extensive neurological impairment and poorer functional outcomes [4].

The patient’s symptoms, progressive bilateral lower limb weakness and urinary incontinence, are consistent with the motor and autonomic dysfunction typically observed in TM [5]. Although the patient exhibited a sensory level suggestive of thoracic involvement (T6), the relatively limited sensory impairment and sparing of the upper limbs rendered the presentation atypical for cervical TM. This diagnostic ambiguity necessitated careful evaluation to rule out alternative etiologies. The differential diagnosis included Guillain-Barré syndrome (GBS), compressive myelopathy, multiple sclerosis (MS), neuromyelitis optica spectrum disorder (NMOSD), and sarcoidosis. However, the presence of upper motor neuron signs, a defined sensory level, no areflexia, and absence of preceding infection made GBS less likely. Comprehensive laboratory workups, including ANA, aquaporin-4 IgG, MOG antibodies, HIV, VDRL, HTLV-1, and vitamin B12 levels, were negative or within normal limits, effectively excluding infectious, nutritional, and autoimmune causes [1].

MRI revealed hyperintense signals on T2-/STIR-weighted sequences from C3 to C6, with focal cord expansion and myelomalacia, radiological features characteristic of inflammatory demyelinating lesions [6]. No mass effect or compressive pathology was identified. Although cerebrospinal fluid (CSF) analysis was not performed due to resource and logistical constraints, the diagnosis of idiopathic transverse myelitis was made based on clinical presentation, exclusion of secondary causes, and consistent imaging findings. We acknowledge the absence of CSF analysis as a limitation in confirming the idiopathic nature of TM; however, the overall clinical-radiologic correlation supported the diagnosis.

Idiopathic TM is believed to involve immune-mediated inflammation of the spinal cord, potentially triggered by subclinical infections or post-infectious immune dysregulation. Parainfectious mechanisms and autoimmune responses may contribute to the pathophysiology, although the exact etiology often remains elusive [7]. High-dose intravenous corticosteroids remain the first-line therapy, aiming to reduce inflammation and limit axonal injury. In this case, methylprednisolone administration resulted in rapid improvement in motor strength and bladder function. Plasma exchange (PLEX) was not initiated, as the patient demonstrated early and significant clinical recovery, in line with current treatment guidelines that reserve PLEX for steroid-refractory cases.

An often underemphasized but critical component of TM management is physiotherapy, which plays a pivotal role in long-term functional recovery. The patient engaged in an early, structured rehabilitation program focusing on muscle strengthening, bladder training, and mobility restoration. At follow-up, she had regained ambulatory function and voluntary bladder control, underscoring the combined benefit of immunotherapy and rehabilitative care.

Although idiopathic TM is typically monophasic, follow-up is essential to monitor for recurrence or evolution into other demyelinating diseases, such as MS or NMOSD. Regular clinical assessments and follow-up imaging may be warranted in selected cases, particularly when atypical features or incomplete recovery are observed [8].

In summary, this case reinforces the importance of maintaining a high index of suspicion for transverse myelitis in patients with acute or subacute neurological deficits, even when symptoms are atypical for the lesion site. Early diagnosis and prompt initiation of corticosteroid therapy, complemented by rehabilitative support, can lead to favorable outcomes, even in anatomically high-risk presentations such as cervical TM.

## Conclusions

This case underscores the importance of maintaining a high index of suspicion for transverse myelitis in patients presenting with acute or subacute neurological deficits, even when the clinical picture is atypical. Cervical involvement, although less common, can manifest primarily with lower limb weakness and autonomic dysfunction, as demonstrated in this patient. Early diagnosis through detailed clinical evaluation and timely MRI, followed by prompt corticosteroid therapy and physiotherapy, played a crucial role in the patient's favorable recovery. The key takeaway from this study is that individualized, guideline-based management, even in resource-limited or non-classical presentations, can significantly improve outcomes in idiopathic transverse myelitis.
